# The Income of Our Hospitals and Dispensaries

**Published:** 1911-06-18

**Authors:** 


### THE INCOME OF OUR HOSPITALS AND DISPENSARIES.

ONCE more Hospital Sunday has come round, and once more a consideration of the sources from which the income of our Metropolitan Hospitals and Dispensaries is raised warrants our taking again as our text: "Londoners give, and give freely, for the benefit of the Sick." The Chancellor of the Exchequer's Scheme of Insurance against Sickness, wide though its effects will be, is only concurrent with, and cannot take the place of, our voluntary system of hospitals. For this reason. Not only do the voluntary hospitals provide for the medical care of the poor when seriously ill, as no attendance in their own homes can possibly do, but they are the training grounds from whence issues that band of trained men and women who fight the battle of the Rich and the Poor alike against the enemy of both—Disease. Without the voluntary hospitals in both phases any scheme of insurance—State supported or otherwise—must fail. Therefore let those who have "Give Freely."

### THE SOURCES OF HOSPITAL INCOME.

Even now, at the end of the first decade of the twentieth century, although we are gradually improving, we Londoners only partly support our Medical Charities of which we, with reason, are prone to boast. We still have to fall back on moneys left by those who are dead in order to pay for the benefits which we ourselves in some form or another receive from the work done by our voluntary medical institutions.

In the Table below we give for each of the ten years 1900 to 1909 the income of the London Hospitals and Dispensaries from all sources except those donations which are given for special purposes, such as building and the like. This Table shows at a glance the amount contributed in each year by Living Londoners, as compared with the amount which, in order to meet the expenditure each year, has to be taken from the contributions of the Dead.

### THE CONTRIBUTIONS OF THE LIVING.

The average Ordinary Income of the London Hospitals and Dispensaries during this ten years has been £1,187,000, towards which Living Londoners have contributed in subscriptions and donations of all kinds; through the King's, Hospital Sunday and Saturday Funds; and by payments for benefits received, £615,000. That is to say, the Living's contribution has been 52 per cent. of the yearly income, or 10s. 5d. out of every sovereign received. Of this sum the payments for benefits received, *i.e.* the payments by patients themselves have averaged about £84,000 a year, or about 9d. for each patient treated either in the Wards or in the Out-patient Departments and Dispensaries. Deducting this £84,000 from the £615,000 contributed by the Living, we have a yearly average income from Londoners of £531,000, or on a population of 4¾ millions a contribution averaging 2s. 3d. a head.

TABLE SHOWING THE SOURCES OF THE INCOME OF THE METROPOLITAN HOSPITALS AND DISPENSARIES IN THE TEN YEARS 1900 TO 1909.

| Year | From the Living | | From the Dead | | | | Total Income |
|---|---|---|---|---|---|---|---|
| | Subscriptions, Donations, Patients' payments, etc. | Percentage of Total | Invested Property | Percentage of Total | Legacies | Percentage of Total | |
| | £ | | £ | | £ | | £ |
| 1900 | 506,051 | 51 | 277,069 | 28 | 208,541 | 21 | 991,661 |
| 1901 | 552,261 | 48 | 283,073 | 25 | 310,975 | 27 | 1,146,309 |
| 1902 | 592,818 | 49 | 269,476 | 23 | 339,091 | 28 | 1,201,385 |
| 1903 | 582,655 | 51 | 258,788 | 22 | 309,093 | 27 | 1,150,536 |
| 1904 | 603,425 | 50 | 258,567 | 22 | 319,303 | 28 | 1,181,295 |
| 1905 | 610,130 | 49 | 264,790 | 21 | 373,914 | 30 | 1,248,834 |
| 1906 | 635,377 | 58 | 272,704 | 25 | 186,284 | 17 | 1,094,365 |
| 1907 | 653,320 | 48 | 296,218 | 22 | 407,918 | 30 | 1,357,456 |
| 1908 | 712,461* | 56 | 290,491 | 23 | 265,493 | 21 | 1,268,445 |
| 1909 | 701,458 | 57 | 294,206 | 24 | 240,523 | 19 | 1,236,187 |
| Average for 10 yrs. | 614,996 | 52 | 276,538 | 23 | 296,113 | 25 | 1,187,647 |

* Includes £72,565, the amount received by the London Hospital in 1908 as the result of its Quinquennial Appeal.

### THE CONTRIBUTIONS OF THE DEAD.

£276,000 has been received on an average each year during the period under review from investments and rents which, as a rule, have resulted from moneys left to the Hospitals by the Dead. This is equivalent to 4s. 7d. out of every sovereign of revenue. Lastly, there has been an average of £296,000 each year, or 5s. in the £, received from the Dead in the form of legacies left during the ten years by former supporters of the medical charities.

Set side by side, therefore, the Living, who enjoy the benefits derived from our voluntary medical charities, have contributed 10s. 5d. as against the 9s. 7d. contributed by the Dead towards each sovereign of the income of the Hospitals.

### What the Medical Charities Expend.

Taking the last year shown in the Table we give above, namely 1909, the Expenditure of the London Hospitals and Dispensaries, exclusive of improvements and extensions, was, in round figures, £1,100,000. Towards this amount there was received as Ordinary Income (exclusive of receipts from invested property and rents) £700,000, or only seven-elevenths of the whole expenditure. To meet the balance recourse had to be made to the income from what had been saved out of the contributions of former benefactors, which amounted to approximately £300,000. This still left a debit balance of £100,000 to be found somehow, and for this the Hospital authorities had to rely on an uncertain source of income, namely, legacies paid to the Hospitals during the then current year. Fortunately for the voluntary institutions, this latter source of revenue did not fail them, and it enabled them, as a whole, to meet their obligations.

Such contributions as these, however, should not be relegated to the Income Account: rather should they be utilised for expenditure on rebuilding where necessary, improvements necessitated by the rapid march of Science, or extensions which have now to be met by special appeals to those who already give largely to the support of our Hospitals.

Cannot we Londoners try to signalise the reign of George V., whose coronation year this is, by endeavouring to put our Metropolitan Voluntary Hospitals on the only sound basis, namely, that of being really supported by voluntary contributions —the voluntary contributions of Living Londoners? In this way we shall not only be honouring our present King, whose interest in our medical charities has been so marked for many years, but we shall be showing our appreciation of the work done by our late beloved King, Edward VII., and be raising up for him a memorial of more lasting effect, and one more after his own heart, than any memorial fashioned by men's hands.

### What Will it Cost Us?

And what a comparatively small individual contribution is necessary to achieve this result!

An extra annual contribution averaging less than 1s. 9d. a head from the inhabitants of London would produce the £400,000 a year which is necessary to place our hospitals in a position to meet their ordinary expenditure without trenching on what should, in part at least, be a reserve fund. Considering that it is estimated that the annual expenditure by Londoners on distilled and fermented liquors is about £18,000,000, or an average on the whole population, men, women and children, of about £4 per head, it only means a diversion of one-forty-sixth part of this sum from our Drink Bill to the hospitals to bring about a result which will enable us to point with pride to an excellent Hospital System, supported by ourselves without any extraneous aid.

